# Antenna-coupled field-effect transistors as detectors for terahertz near-field microscopy

**DOI:** 10.1039/d0na00928h

**Published:** 2021-02-12

**Authors:** Matthias M. Wiecha, Rohit Kapoor, Alexander V. Chernyadiev, Kęstutis Ikamas, Alvydas Lisauskas, Hartmut G. Roskos

**Affiliations:** Physikalisches Institut, Johann Wolfgang Goethe-Universität Max-von-Laue-Straße 1 60438 Frankfurt am Main Germany wiecha@physik.uni-frankfurt.de roskos@physik.uni-frankfurt.de; CENTERA Laboratories, Institute of High Pressure Physics PAS 01-142 Warsaw Poland; Institute of Applied Electrodynamics and Telecommunications, Vilnius University 10257 Vilnius Lithuania; The General Jonas Žemaitis Military Academy of Lithuania 10322 Vilnius Lithuania

## Abstract

We report the successful implementation of antenna-coupled terahertz field-effect transistors (TeraFETs) as homodyne detectors in a scattering-type scanning near-field optical microscope (s-SNOM) operating with radiation at 246.5 GHz. The devices were fabricated in Si CMOS foundry technology with two different technologies, a 90 nm process, which provides a better device performance, and a less expensive 180 nm one. The high sensitivity enables s-SNOM demodulation at up to the 10th harmonic of the cantilever's oscillation frequency. While we demonstrate application of TeraFETs at a fixed radiation frequency, this type of detector device is able to cover the entire THz frequency range.

## Abstract 2

We report the successful implementation of antenna-coupled terahertz field-effect transistors (TeraFETs) as homodyne detectors in a scattering-type scanning near-field optical microscope (s-SNOM) operating with radiation at 246.5 GHz.

## Graphical abstract

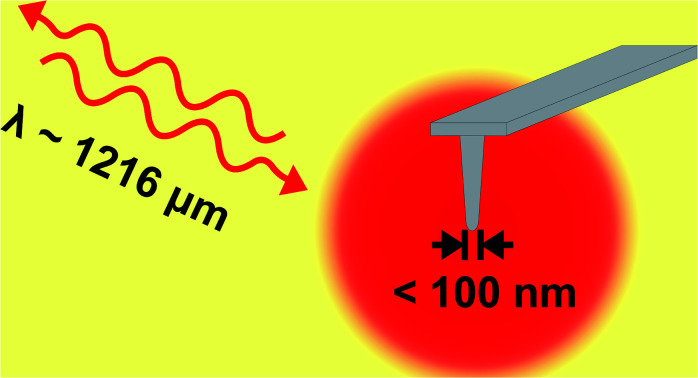

## 1 Introduction

Free-space terahertz (THz) and sub-THz spectroscopy and imaging suffer from a poor spatial resolution due to the Abbe diffraction limit. To overcome this limitation, several techniques have been developed, amongst them scattering-type scanning near-field optical microscopy (s-SNOM), a near-field technique which offers nanometer-scale spatial resolution largely independent of the wavelength of the radiation used.^1^ One big challenge of s-SNOM is the weak signal strength received at the detector, which makes its usage challenging especially in the THz and sub-THz regime with its large wavelength and the concomitant large size of the illuminated sample spot, and with its lack of powerful sources. Different ways have been found to deal with this challenge. One approach is the use of pulsed terahertz time-domain spectroscopy together with electro-optical sampling,^2,3^ another one is the use of long-pulse or continuous-wave radiation from high-power sources such as THz gas lasers^4^ and free electron lasers^5^ in combination with bolometric detection at low temperature. The more challenging task is indeed the detection, which has to be fast enough to resolve the periodic vertical cantilever movement occurring with a typical frequency *Ω* in the range of tens to hundreds of kHz. At frequencies above 1 THz, where quantum cascade lasers (QCLs) are available, one can use QCLs not only as emitters, but also as detectors, even in this way that only a single device is employed having both roles at once (the radiation back-scattered from the s-SNOM's tip is fed into the laser cavity).^6^ For measurements at sub-THz frequencies, several publications have now reported the use of Schottky diodes as detectors.^7–9^ They are typically operated in combination with narrow-band multiplier-based electronic sources, which permits heterodyne detection by mixing the back-scattered radiation with that from a second source frequency-locked to the first one with a frequency offset *Δ* for demodulation at sidebands *Δ* ± *nΩ*, *n* = 1, 2, 3, …^7,9^ The state-of-the-art technology for coherent (field- and phase sensitive) s-SNOM detection in the infrared and visible spectral range is based on the so-called pseudo-heterodyne detection scheme.^10,11^ It is an interferometric technique using continuous (usually periodic) mirror displacement in one arm of an interferometer in order to achieve phase sensitivity. Given the large wavelength in the sub-THz frequency range, the application of this technique is impractical at those frequencies because of the need for large mirror displacements. One would need about 120× higher displacement amplitudes at 246.5 GHz compared to standard mid-IR s-SNOM at the wavelengths of CO_2_-lasers, which is technically so demanding that – to our knowledge – it has not been implemented by any group so far. The heterodyne scheme addressed above is an alternative, which comes, however, at the cost of a second radiation source. In this work, we employ homodyne interferometric detection as yet another alternative for sub-terahertz s-SNOM sensing.^12^ It offers the interferometric signal enhancement of the pseudo-heterodyne technique (in our case about 50× higher signals in comparison to non-interferometric detection), phase sensitivity by two subsequent measurements with different mirror settings, and it has the option for samples, which do not introduce an optical phase shift, to record the near-field signal in a single measurement by setting the interferometer to the maximum-signal position.

Here, we introduce s-SNOM detection by field-effect transistors with monolithically integrated antennas for THz frequencies (TeraFETs). They are operated at room temperature. This type of detector of GHz and THz radiation is on the verge of becoming a mainstream device rivalling diode-based detectors. The detection mechanism is distributed resistive mixing in the transistor's channel,^13–15^ enhanced by plasma-wave phenomena at high frequencies as predicted in ref. 16. As the FET is operated without source-drain bias voltage, it exhibits only thermal noise.^17^ The detectors can be fabricated in many material systems, but devices made in the Si CMOS technology continue to exhibit the best performance in terms of the noise-equivalent power values achieved,^18^ only recently rivalled by detectors made in AlGaN/GaN technology.^19,20^ Detectors can be built for discrete frequency bands high in the THz regime^21,22^ or as single devices with large detection bandwidth and flat frequency response, *e.g.* covering 0.1–2.2 THz in ref. 23. With an ultrafast response on the time scale of the inverse cut-off frequency *f*_T_ (in the range of tens to hundreds of GHz), the devices can be employed as heterodyne receivers.^18,24,25^ As such, they are being used for imaging applications with depth resolution^26^ and for full-scale three-dimensional imaging.^27^

The devices employed for this study (see Fig. 2, to be discussed below) were not specifically designed for the task. While they exhibit a novel chip layout, their performance parameters are on a par with those obtained with other TeraFETs developed by us and others, and could be replaced by those. We investigate two devices, fabricated with different foundry technologies, and compare their performances with each other. One device is based on a state-of-the-art 90 nm CMOS process for best performance, the other on a 180 nm CMOS process (of the same foundry). The latter is interesting from a commercial point of view as it employs rather relaxed design rules (indicated by the large gate length),^14,28,29^ which both keeps the fabrication costs comparatively low and is favorable for the electrical robustness (including stability against electrostatic shock) of the device.

## 2 Experimental setup

**Fig. 1:**
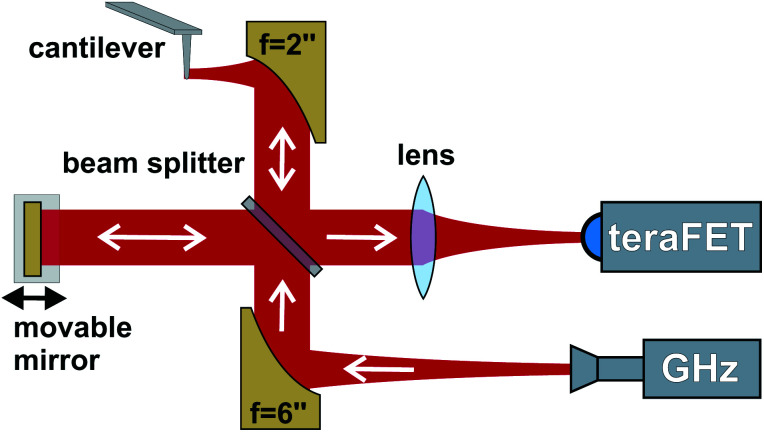
Interferometric setup for the homodyne near-field measurements. Radiation propagating along the signal arm of the interferometer is focussed onto the cantilever, the backscattered light is directed onto the TeraFET detector. The reference arm with the movable mirror enhances the signal level on the detector if the mirror is positioned for constructive interference. Phase changes by the sample-under-test are identified by measurements at various mirror positions.

The setup with the interferometer for homodyne detection is sketched in Fig. 1. As usual for s-SNOM, the radiation is focussed onto the oscillating tip apex of the cantilever of an atomic force microscope (AFM, cantilever oscillation frequency: *Ω*). The sample-under-test on a translation stage is scanned under the tip. Radiation, backscattered both as a consequence of the near-field interaction between the tip and the sample, and by any other scattering object in the beam path is detected by the TeraFET whose signal is fed to a lock-in amplifier. The unwanted, but dominant far-field contribution in the signal is suppressed by higher-harmonic demodulation at *nΩ*, *n* = 1, 2, 3, …, in order to allow the extraction of the local near-field properties of the sample (*i.e.* its dielectric function).^9,30^ Simultaneously, the sample's topography is mapped by the AFM functionality. The AFM is a home-built device^11^ controlled with an Anfatec Instruments AG DS4L SPM-controller and it is based on a “scanning sample” design. For the s-SNOM operation, we employ a radiation source based on a microwave synthesizer followed by an amplifier, a multiplier chain and a horn antenna (all three from RPG – Radiometer Physics GmbH). It provides linearly polarized free-space radiation at 246.5 GHz with an output power of 75 μW. The exact frequency was chosen by frequency fine-tuning for the best signal of the lock-in amplifier, which strongly depends on the beam-splitting ratio (see below) and varies upon frequency changes as a consequence of the Fabry–Perot resonances within the beam splitter.

The emitted radiation is collimated by a paraboloidal mirror (*f* = 6′′, diameter *d* = 2′′) and focussed by a second paraboloidal mirror (*f* = *d* = 2′′, mounted on a 3D translation stage) onto the apex of the probe tip (Rocky Mountain Nanotechnology RMN tip model RMN-25Pt300B, tip length: 80 μm) oscillating at a frequency *Ω* = 21.5 kHz (despite the use of the letter *Ω* not an angular frequency). Due to its length, this type of probe tip exhibits a better THz response than standard probes used for s-SNOM in the infrared.^31^ A beam splitter made from high-resistivity silicon (uncoated, thickness: 2 mm) enables generation of a reference beam for the interferometric detection, and to guide a large part of the backscattered radiation to the detector. The beam splitter's power splitting ratio at the chosen radiation frequency is 40 : 60, the larger part of the radiation from the emitter going to the reference beam.

**Fig. 2:**
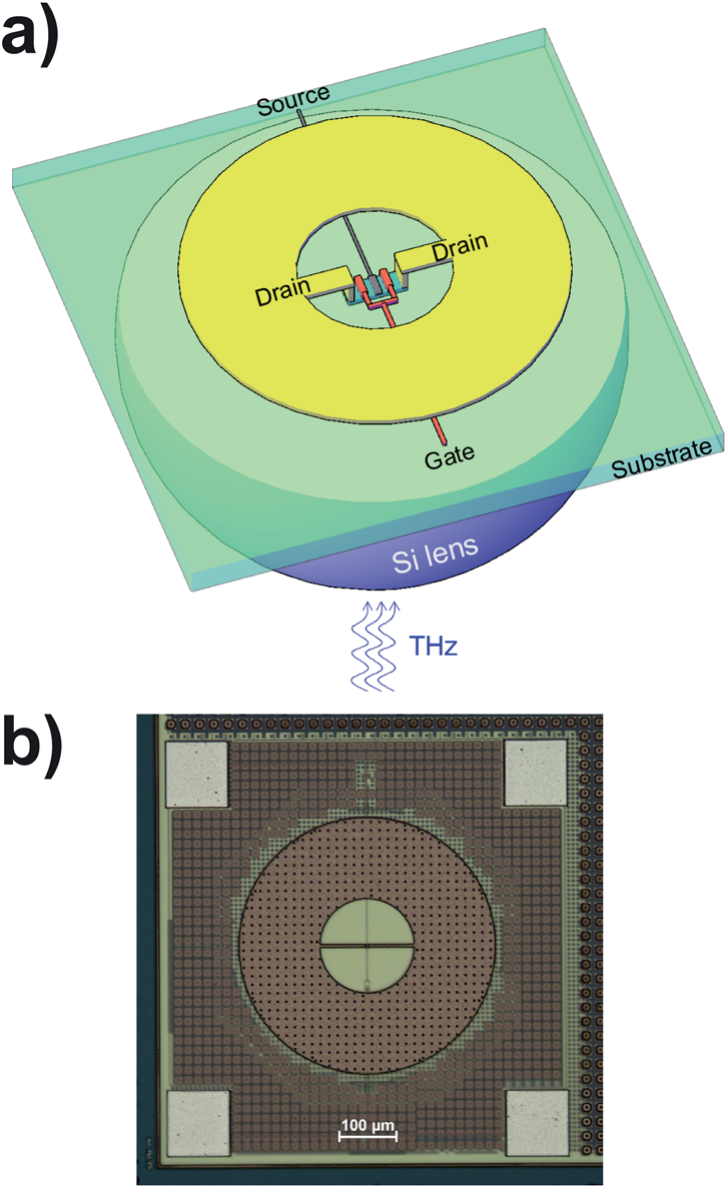
(a) Schematic and (b) real image of the Si CMOS TeraFET detectors. The dimensions of the antenna metallization (yellow) and of the substrate lens (violet) in (a) are not to scale in order bring out the transistor structure in a clear manner. The annular antenna is the brighter brown structure in (b), while the dark brown area around it consists of metal patches which have no antenna functionality at the target frequency range; they had to be placed into the detector design in order to obey the design rules of the foundry which demand a certain metal coverage in order to prevent the build-up of excessive mechanical stress.

The two Si CMOS TeraFET detectors investigated here were both fabricated at the TSMC foundry, Taiwan, but with different technology nodes, 90 nm and 180 nm, respectively. In the following, the devices are denoted accordingly as either 90 nm or 180 nm detector. Both are based on the same design concept developed by some of us. Each detector consists of an annular ring antenna with an integrated dipole, which also acts as an impedance transformer, and a dual-finger transistor with a gate length of 90/180 nm and a gate width of 1 μm. A 3D scheme of the layout of the device is shown in Fig. 2a (the dimensions are not to scale), and a microscope photograph with a top view of one of the actual devices is displayed in Fig. 2b. Note that the drain terminals are electrically connected only *via* the antenna metallization. Unlike annular ring microstrip antennas or other patch antennas,^28^ the design is based on a ground-plane-free approach in order to enable in-coupling of the THz radiation from the substrate side. This allows us to easily apply a hyperhemispherical substrate lens made from high-resistivity single-crystalline silicon (diameter: 12 mm) as schematically shown in Fig. 2a. The backside in-coupling comes at the penalty of moderate free-carrier-induced absorption losses of the THz radiation in the 280 μm-thick silicon substrates of the detector chips, which are lightly *p*-doped (specific resistance as specified by the foundry: 10 Ω cm, which corresponds to a carrier density of 1.25 × 10^15^ cm^−3^). The absorption losses lead to a power attenuation of the THz radiation by about 26%.

**Fig. 3:**
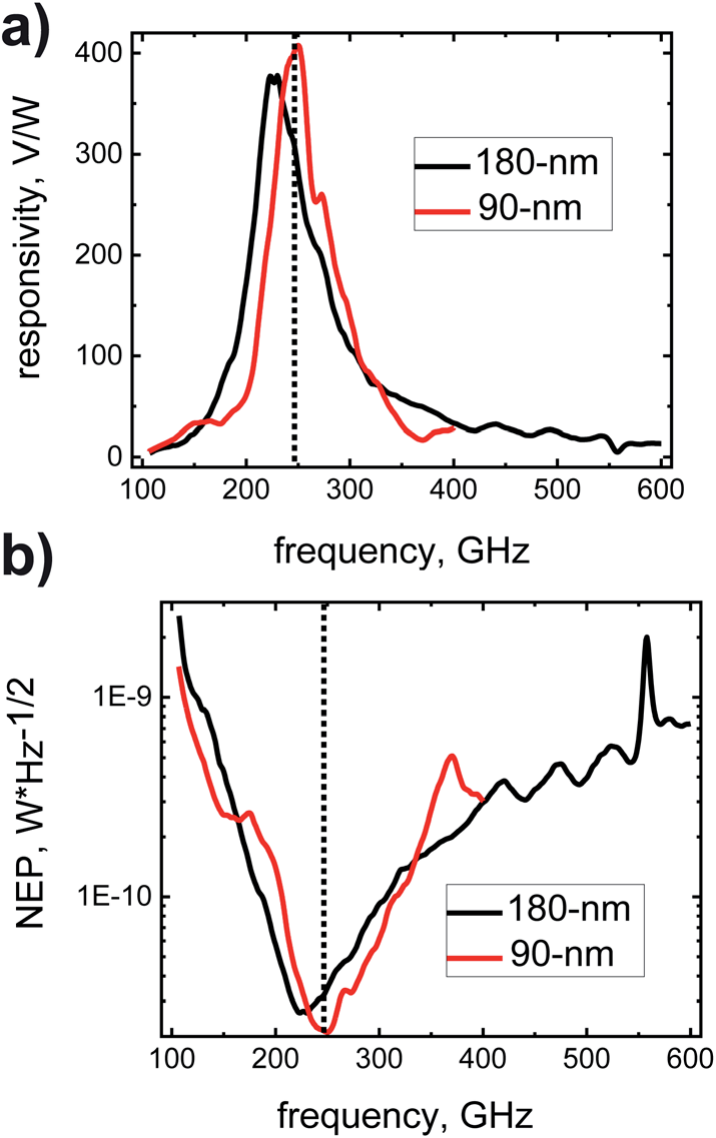
(a) Voltage responsivity and (b) noise-equivalent power (NEP) of the detector devices. The vertical dotted lines indicate the radiation frequency of 246.5 GHz.

A detailed performance characterization of a 90 nm device^32^ is found in ref. 33. The red curve in Fig. 3 displays the voltage responsivity and the noise-equivalent power (NEP) of the present detector. The responsivity peaks at a value of 408 V W^−1^, while the best NEP value is 21 pW Hz^−1/2^. These optimal values are reached at 250 GHz, near the operation frequency of our radiation source, where the responsivity is 402 V W^−1^ and the NEP remains at 21 pW Hz^−1/2^. The 180 nm detector exhibits a responsivity of 380 V W^−1^ and a noise-equivalent power of 27 pW Hz^−1/2^ at 225 GHz, the frequency of best performance, with the corresponding values at the operation frequency of the s-SNOM being 308 V W^−1^ and 32 pW Hz^−1/2^, respectively, see the black curve in Fig. 3. In both cases, the gate bias voltage was 0.55 V, and – as usual for TeraFETs – no source-drain bias voltage was applied in order to operate at the best noise performance.

During the s-SNOM measurements, the rectified output signal of the TeraFET is fed into a lock-in amplifier (Zurich Instruments MFLI) for higher-harmonic demodulation. In the experiments described in the following, the position of the mirror in the reference arm remained fixed at a position of constructive beam interference, found by maximizing the 1*Ω* s-SNOM signal at the beginning of the measurement on a metallic region of the respective sample-under-test.

## 3 Results and discussion

**Fig. 4:**
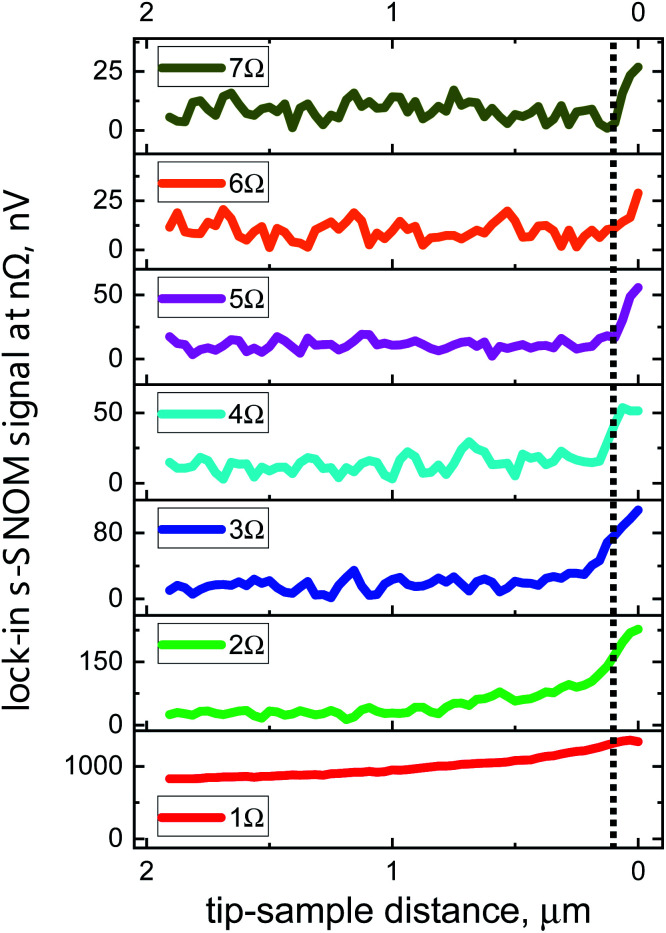
Approach curves recorded with a Au film as sample-under-test with the 180 nm detector. The curves were recorded at different demodulation frequencies: at the fundamental tip oscillation frequency 1*Ω* and at different harmonics *n* from 2*Ω* to 7*Ω*. The dotted line at 100 nm serves as a guide to the eye to facilitate visual distinction of the signal rise upon approach of null distance.

**Fig. 5:**
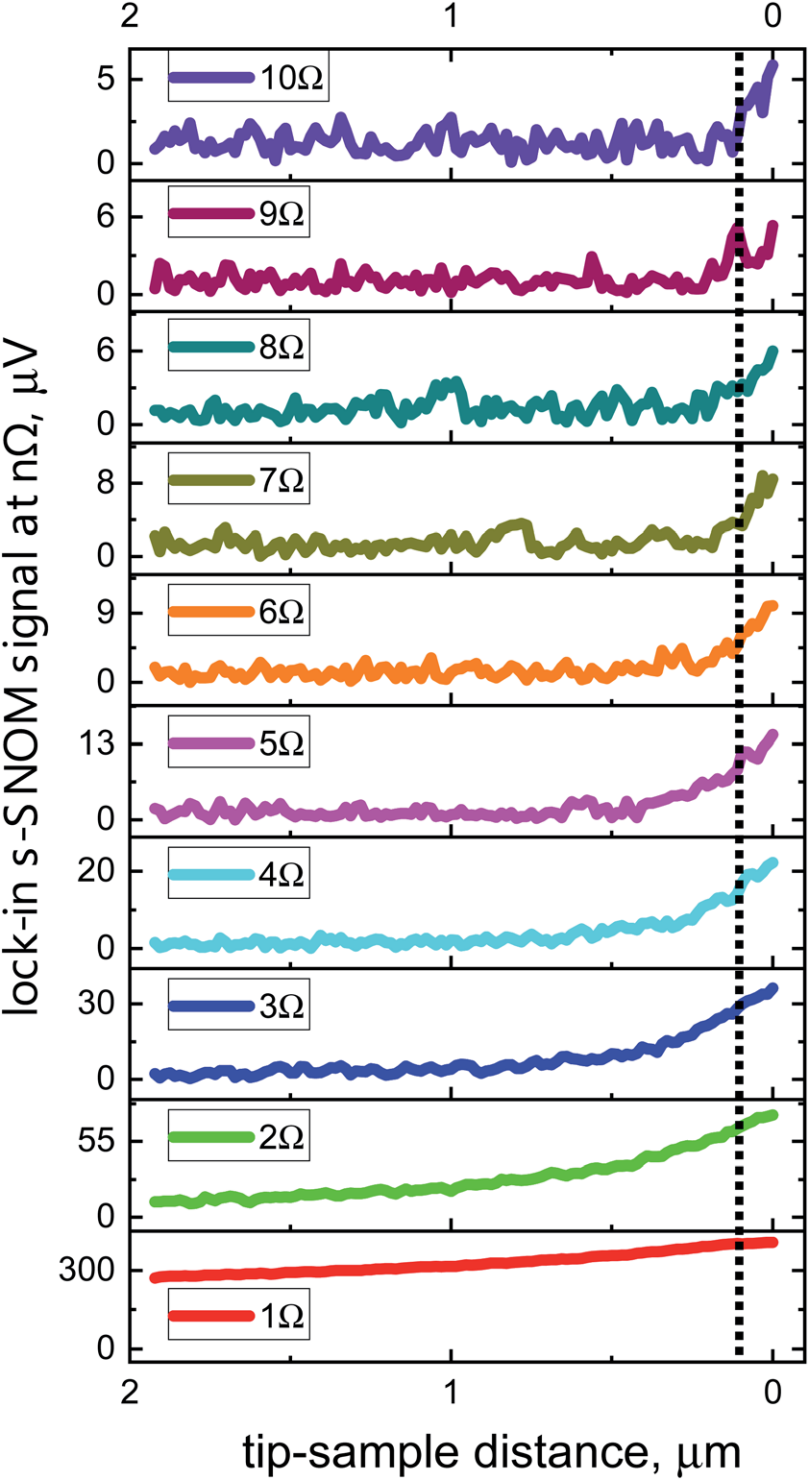
Approach curves at different harmonics from 1*Ω* up to 10*Ω* measured with the 90 nm detector. The dotted line is placed at a distance of 100 nm, *cf.*Fig. 4.

Fig. 4 and 5 display so-called approach curves at various demodulation frequencies for the two detectors. The curves were recorded with a Au-on-Si sample by measuring the s-SNOM signal as a function of the distance between tip apex and sample upon retraction of the tip. All data represent single distance scans and were measured with the same integration time constant *t*_c_ = 100 ms of the lock-in amplifier. The slow decay of the 1*Ω* s-SNOM signal with increasing distance indicates that there are strong background contributions present at the fundamental tip oscillation frequency, whereas the signals at higher harmonics (*n* ≥ 2) continue to decay more rapidly with rising distance as the order number *n* increases, revealing increasingly stronger localization of the respective electric fields at the apex region of the tip. In addition, one clearly notices the increase of the noise level with the order of the harmonics which is a consequence of the decrease of the signal-to-noise ratio (SNR) with rising value of *n*. The high sensitivity of the 90 nm detector allows to obtain useful signals up to *n* = 10.

Table 1 specifies SNR values obtained with both devices from these and other approach-curve measurements. The table lists values for a time constant of the lock-in amplifier of *t*_c_ = 1 s; for the 90 nm device, we also present SNR values for *t*_c_ = 100 ms, because it is common in the literature to provide noise-related performance data such as SNR and NEP values for this integration time. The first value in the table is for a freely oscillating tip away from a sample surface, the signal being that back-scattered from the tip upon standard illumination alignment and recorded at 1*Ω*. The other values are for the tip interacting with the Au surface (same beam alignment), recorded at the various demodulation frequencies 1*Ω* to 10*Ω*. The 90 nm TeraFET exhibits a 3–5× better SNR than the 180 nm device, and reaches a performance level which is comparable to (or even better than) that reached with Schottky diodes as detectors, for which one finds SNR values > 10 for demodulation at 2*Ω* in ref. 9, and values of 14 at 2*Ω*, respectively 7 at 3*Ω*, in ref. 7, in measurements on Au/highly doped Si for *t*_c_ = 100 ms. Also, s-SNOM systems measuring with pulsed THz radiation, generated and detected optoelectronically with femtosecond laser pulses, do not seem to reach better SNR values (SNR of >20, respectively >14, at 2*Ω* reported in ref. 34 and 35] on graphene for *t*_c_ = 100 ms).

SNR values obtained for radiation back-scattered from a freely oscillating tip and recorded at the fundamental frequency 1*Ω* (“1*Ω* (free)”) in comparison with the SNR values found in s-SNOM measurements with demodulation at *nΩ* for a tip in interaction with a Au surface (“*nΩ* (s-SNOM)”). Values are for a time constant of *t*_c_ = 1 s of the MFLI lock-in amplifier for both detectors. In addition, the lower line presents values for *t*_c_ = 100 ms for the 90 nm detector

**Table:** 

SNR	1*Ω* (free)	1*Ω* (s-SNOM)	2*Ω* (s-SNOM)	3*Ω* (s-SNOM)	4*Ω* (s-SNOM)
180 nm (1 s)	53	108	39	10	12
90 nm (1 s)	388	387	49	49	39
90 nm (100 ms)	58	78	18	10	8

**Table:** 

5*Ω* (s-SNOM)	6*Ω* (s-SNOM)	7*Ω* (s-SNOM)	8*Ω* (s-SNOM)	9*Ω* (s-SNOM)	10*Ω* (s-SNOM)
—	—	—	—	—	—
27	15	12	8	8	5
4.6	4.0	2.9	2.1	1.7	1.4

In the following, we test the application of the TeraFET detectors in typical s-SNOM measurement situations.

**Fig. 6:**
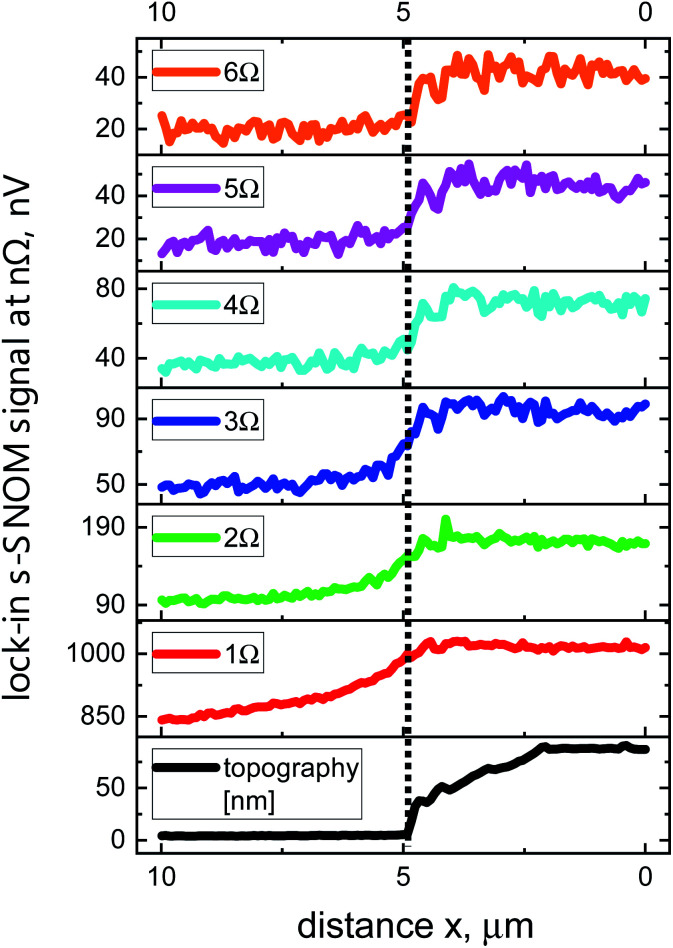
Line scans across a Si–Au step (Si: left, Au: right). The data were measured with the 180 nm detector. The black line in the panel at the bottom represents the AFM-recorded topography, while the colored lines are s-SNOM data obtained at various demodulation frequencies 1*Ω*–6*Ω*. The vertical dotted line marks the boundary between the two materials.

Sensitivity to different materials is demonstrated in Fig. 6 which display line scans across an edge of an Au patch on a Si substrate. The data were recorded with a lock-in integration time of *t*_c_ = 61.15 ms, 10 scans were averaged. The s-SNOM signal is stronger on the Au surface than on the Si surface. The 1*Ω* curve shows the step only with a weak contrast because of the dominance of the strong background signal. For higher-harmonic demodulation, the signal change at the step is much more pronounced, with barely any difference in abruptness between the curves for the high harmonics.

**Fig. 7:**
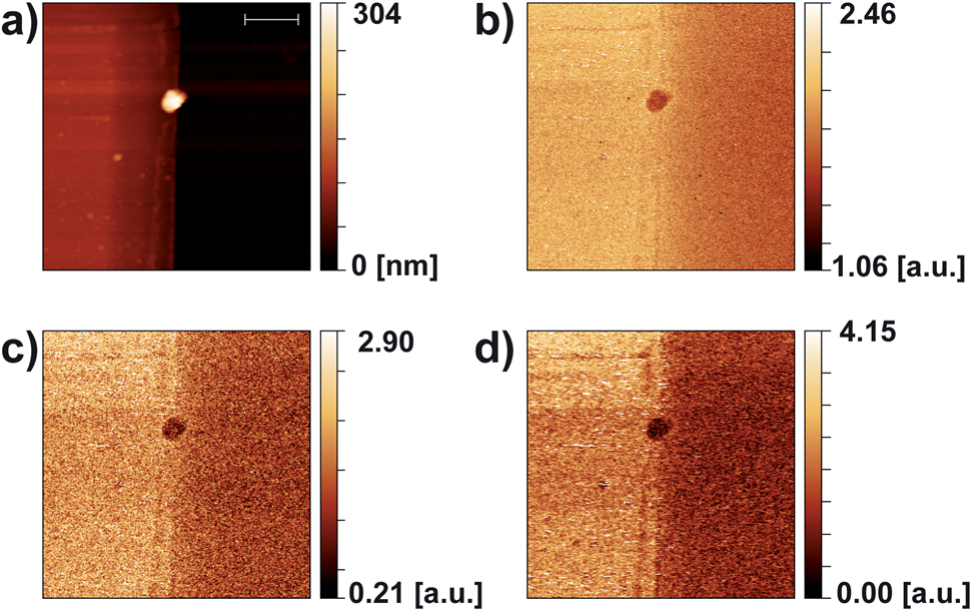
Large-area images at a Au–Si edge with a dirt particle sticking to the edge (length of scale bar in (a): 2 μm). Au is on the left side, Si on the right. (a) Topography, (b–d) 1*Ω* to 3*Ω* s-SNOM maps taken with the 180 nm TeraFET detector. Noise levels of the s-SNOM maps are not directly comparable as the 1*Ω* and 2*Ω* signals were recorded with a 7265 Dual Phase DSP lock-in amplifier, the 3*Ω* signal in parallel with the MFLI lock-in amplifier which has a better SNR. Integration time constant: 20 ms, time needed for recording the full image: 1.5 h.

Fig. 7 even more clearly highlights the ability to distinguish materials even when working only at a single radio frequency. The measurement was again taken over an Au–Si edge (Au on the left side) on which a dirt particle was found to stick to the edge. The three types of materials yield different height profiles (Fig. 7a), but also are discernible by different s-SNOM scattering strengths as seen for all demodulation frequencies 1*Ω* to 3*Ω* (Fig. 7b–d).

**Fig. 8:**
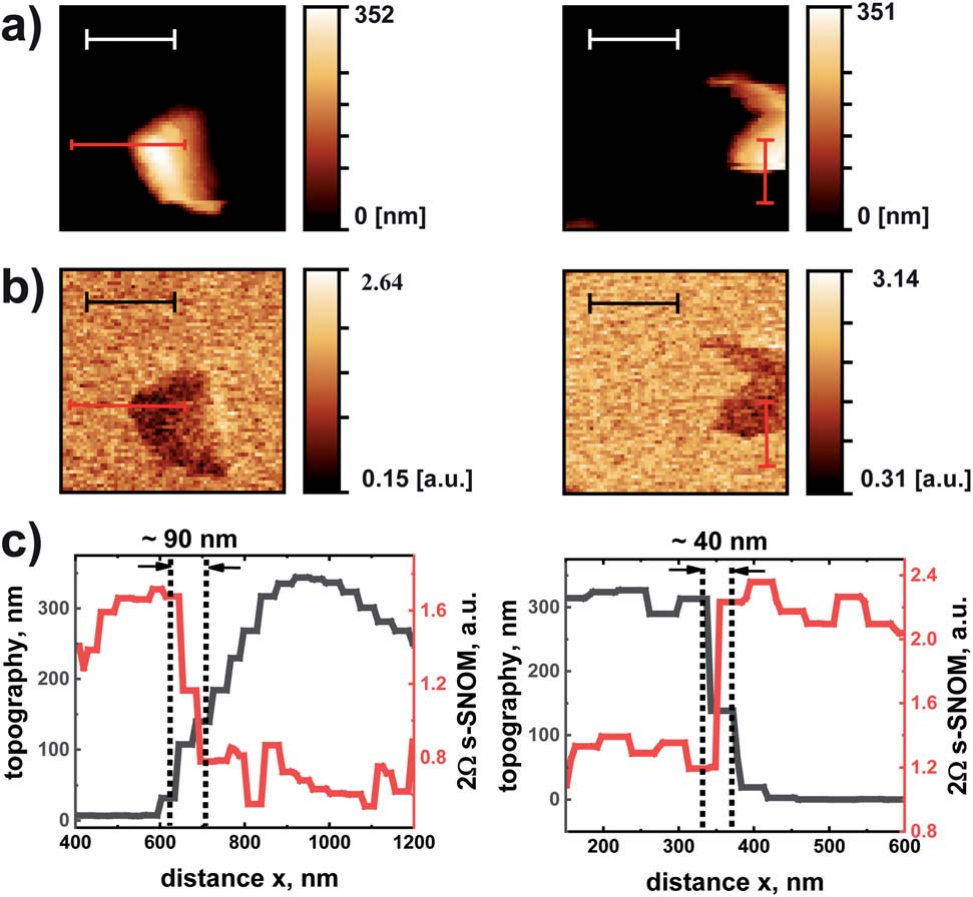
(a) AFM-topography maps of two regions of a Si surface with dielectric islands on the sample (length of white scale bar: 1 μm). The line scans were taken with motion of the tip in horizontal direction and raster translation for each following scan in vertical direction. (b) Simultaneously recorded 2*Ω* s-SNOM images (length of black scale bar: 1 μm, *t*_c_ = 61.15 ms). The measurements were made with the 180 nm detector. (c) AFM and 2*Ω* s-SNOM line profiles along the red lines shown in (a) and (b). The AFM data (with values on the left *y*-axis) are plotted in grey color, the 2*Ω* s-SNOM data (right *y*-axis) in red color. The width of the end bars of the red lines in the images shown in (a) and (b) indicate the spatial range over which the line-scan data of (c) were averaged. The vertical dotted black lines demarcate the distance range of the step-like change of the s-SNOM signal.

Another typical measurement which we reproduced with one of the TeraFET detectors is a basic test of the lateral spatial resolution. For an evaluation of the resolution which can be reached with an s-SNOM, a metal edge on an insulating dielectric substrate is not a suitable object (as is the case for any material with a strong near-field interaction with the tip at the given wavelength of the radiation).^36^ For Au, which is a near-perfect conductor at THz frequencies (no excitation of plasmon polaritons), the interaction with the tip is expected to strongly vary as the tip moves across the edge and the local field distribution strongly changes. This is different for edges of dielectric films with their weaker tip interaction. We therefore performed measurements on dielectric surface contaminations (patches of residual photoresist) on the Si layer. Fig. 8a shows AFM topography maps of two such sample regions, Fig. 8b the simultaneously recorded 2*Ω* s-SNOM images. The amplitude of the 2*Ω* s-SNOM signal is higher on the Si surface than on the resist residue. Fig. 8c displays profiles of the topography and the 2*Ω* s-SNOM signal along the red lines shown in the maps. The lines are oriented along (left figure) and perpendicular (right figure) to the horizontal scan direction of the probe tip. The 2*Ω* s-SNOM signal changes in both cases abruptly at the edges of the dielectric material, the signal change occurring over a scan distance of less than 100 nm, with the smallest distance found to be 40 nm. While we do not identify this distance as the resolution limit of the measurement system in the strict sense, as one should avoid or at least minimize topography differences when determining the lateral resolution, the data show that one obtains good contrast with the TeraFET detectors in such type of measurements.

**Fig. 9:**
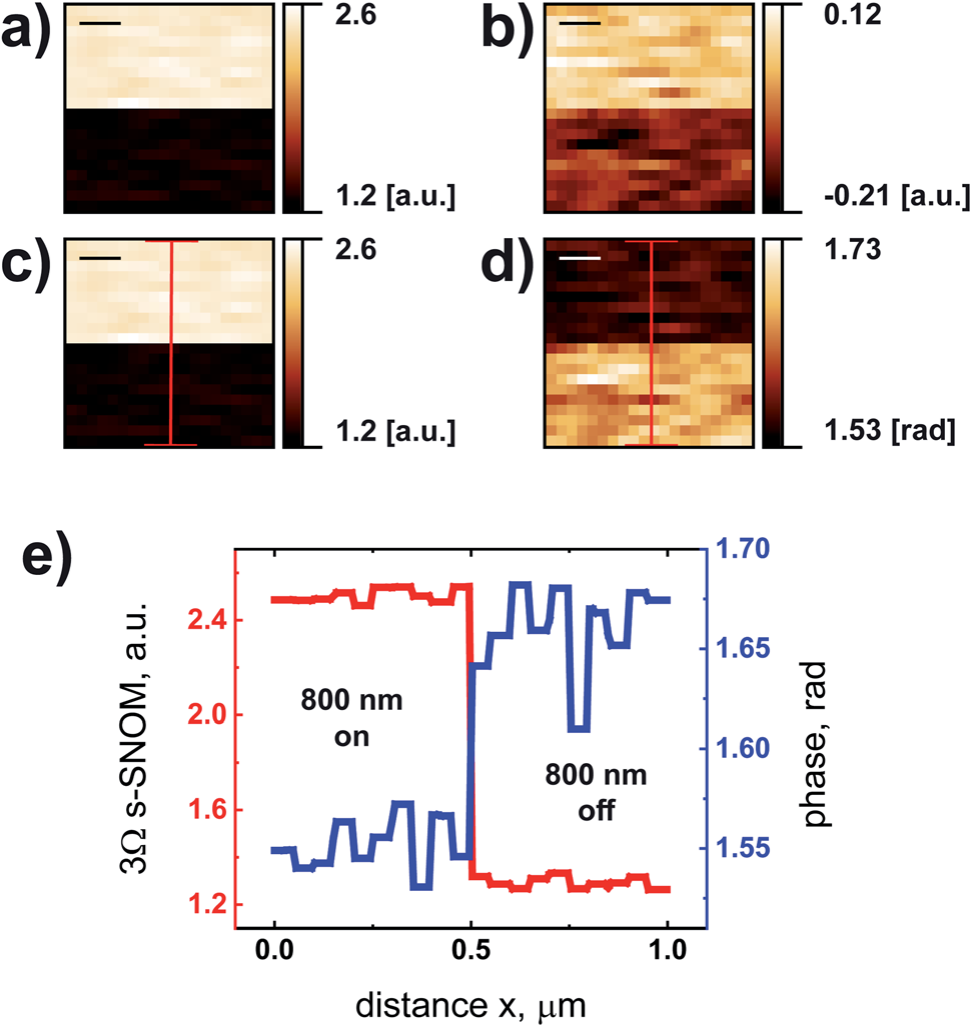
(a and b) Consecutive 3*Ω* homodyne s-SNOM measurements at *f* = 246.5 GHz with shift of the reference phase by mirror displacement. After half of each areal scan, the exciting 800 nm laser light was blocked (top half of each panel in (a) to (d): light on; bottom half: light off). (c) Optical amplitude and (d) optical phase calculated from the raw data displayed in (a) and (b) (for details, see main text). (e) Line profiles across the near-field maps (along the red lines in (c) and (d)) reveal the measured amplitude and phase contrast. Scale bars: 200 nm, lock-in integration time: *t*_c_ = 1 s, all measurements made with the 90 nm detector.

We finally verified that we obtain the phase sensitivity of homodyne detection^1^ with our detectors, and demonstrate this with s-SNOM measurements on photo-excited silicon. The results are displayed in Fig. 9. The specimen was a polished Si wafer (thickness: 500 μm, 〈100〉-cut, vendor: University Wafer Inc.) with weak B-doping (specific resistance: 10–20 Ω cm). The wafer was illuminated by continuous-wave laser light at a wavelength *λ* of 800 nm from a Ti:sapphire laser. The laser radiation was fiber-coupled and focused by a lens with a focal length of 10 cm onto the sample in the region of the probe tip of the s-SNOM, where it generated an electron–hole plasma whose Drude response modified amplitude and phase of the s-SNOM signal of the semiconductor.^7,37^ The laser power was adjusted to maximize the phase contrast.

Fig. 9a and b show two signal maps recorded by consecutive 3*Ω* s-SNOM measurements at *f* = 246.5 GHz, the data taken with the same sensitivity and phase settings of the lock-in amplifier.^1^ Between those measurements, the mirror of the reference arm was translated by a distance of 
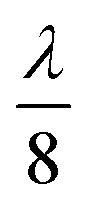
 which corresponds to a total shift of the optical phase by 
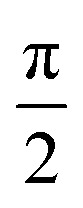
. Fig. 9a hence displays the lock-in signal *r*_1,3*Ω*_ = *s*_3*Ω*_ cos(*ϕ*_3*Ω*_), Fig. 9b the corresponding signal *r*_2,3*Ω*_ = *s*_3*Ω*_ sin(*ϕ*_3*Ω*_). *ϕ*_3*Ω*_ is the optical phase which includes propagation effects of the scattered radiation as well as a phase change by the interaction of the radiation with the specimen-under-test. In order to demonstrate the latter in our experiment, the illumination light was blocked after half of each s-SNOM scan, such that the upper half of each image shows the s-SNOM response of laser-illuminated Si, the lower half that of the un-illuminated Si.

From the lock-in signals *r*_1,3*Ω*_ and *r*_2,3*Ω*_, one calculates amplitude *s*_3*Ω*_ and phase *ϕ*_3*Ω*_ of the scattered near-field signal, using the equations 
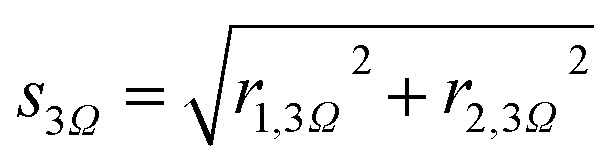
 and *ϕ*_3*Ω*_ = arctan2(*r*_1,3*Ω*_,*r*_2,3*Ω*_), respectively. Fig. 9c displays the resulting amplitude map, Fig. 9d the phase map. One clearly sees the effect of the photo-excited charge carriers on both the amplitude and phase of the scattered radiation. The changes are presented also in the line profiles in Fig. 9e, taken along the red lines in Fig. 9c and d.

## 4 Conclusions

In summary, we demonstrated the applicability of TeraFET detectors based on antenna-coupled field-effect transistors for near-field s-SNOM microscopy at (sub-)terahertz frequencies. We employed homodyne detection whose phase sensitivity provides access to the imaginary part of the dielectric function of materials. The high responsivity of the detectors allowed demodulation of the s-SNOM signal even at the tenth harmonic of the cantilever oscillation frequency.

Unlike microwave imaging, which operates with waveguide coupling of the radiation to the probe tip,^38^ the presented approach with free-space radiation and employing antenna coupling to the detector, is free from waveguide-defined frequency band limitations and can be applied for seamless broad-band spectroscopy over the entire sub-THz and THz frequency regime (0.1–10 THz) with sub-100 nanometer resolution. Similar to Schottky diodes^39^ and other kinds of diode-based detectors, TeraFETs can be employed as both power detectors and heterodyne receivers. Their performance can be enhanced significantly by cooling them to cryogenic temperatures.^40–42^ The use of compact antenna-coupled diode or TeraFET detectors (equipped usually with substrate lenses) opens the possibility for placing the detectors – or arrays of them – close to the probe tip, which may enhance the radiation coupling efficiency, but specifically can be exploited to detect radiation patterns and investigate emission profiles of the specimen.

With this work, we contribute to the development of THz and sub-THz s-SNOM nanoscopy which is – among other potential applications – very well suited to probe the conductivity of semiconductors and of new materials for future electronic devices on the nanoscale.

## Author contributions

M. M. W. prepared the s-SNOM setup, performed and analyzed the measurements and wrote the draft of the manuscript; R. K. performed s-SNOM measurements and analyzed data; A. V. C. characterized the optical response of the detector; K. I. developed its layout; A. L. designed the detector, managed its fabrication and packaging, and advised on its electrical and optical handling; H. G. R. planned and supervised the project and wrote the manuscript. All authors contributed in the finalization of the paper.

## Conflicts of interest

There are no conflicts of interest to declare.

## Supplementary Material

## References

[cit1] Taubner T., Hillenbrand R., Keilmann F. (2003). J. Microsc..

[cit2] Stinson H. T., Sternbach A., Najera O., Jing R., Mcleod A. S., Slusar T. V., Mueller A., Anderegg L., Kim H. T., Rozenberg M., Basov D. N. (2018). Nat. Commun..

[cit3] von Ribbeck H. G., Brehm M., van der Weide D. W., Winnerl S., Drachenko O., Helm M., Keilmann F. (2008). Opt. Express.

[cit4] Huber A. J., Keilmann F., Wittborn J., Aizpurua J., Hillenbrand R. (2008). Nano Lett..

[cit5] Soltani A., Kuschewski F., Bonmann M., Generalov A., Vorobiev A., Ludwig F., Wiecha M. M., Čibiraitė D., Walla F., Winnerl S., Kehr S. C., Eng L. M., Stake J., Roskos H. G. (2020). Light: Sci. Appl..

[cit6] Degl'Innocenti R., Wallis R., Wei B. B., Xiao L., Kindness S. J., Mitrofanov O., Braeuninger-Weimer P., Hofmann S., Beere H. E., Ritchie D. A. (2017). ACS Photonics.

[cit7] Liewald C., Mastel S., Hesler J., Huber A. J., Hillenbrand R., Keilmann F. (2018). Optica.

[cit8] Dai G. B., Geng G. S., Zhang X. X., Wang J., Chang T. Y., Cui H.-L. (2019). IEEE Access.

[cit9] Chen X. Z., Liu X., Guo X. D., Chen S., Hu H., Nikulina E., Ye X. L., Yao Z. H., Bechtel H. A., Martin M. C., Carr G. L., Dai Q., Zhuang S. L., Hu Q., Zhu Y. M., Hillenbrand R., Liu M. K., You G. J. (2020). ACS Photonics.

[cit10] Ocelic N., Huber A., Hillenbrand R. (2006). Appl. Phys. Lett..

[cit11] Walla F., Wiecha M. M., Mecklenbeck N., Beldi S., Keilmann F., Thomson M. D., Roskos H. G. (2018). Nanophotonics.

[cit12] Maissen C., Chen S., Nikulina E., Govyadinov A., Hillenbrand R. (2019). ACS Photonics.

[cit13] Knap W., Teppe F., Meziani Y., Dyakonova N., Lusakowski J., Boeuf F., Skotnicki T., Maude D., Rumyantsev S., Shur M. (2004). Appl. Phys. Lett..

[cit14] Lisauskas A., Pfeiffer U., Öjefors E., Haring Bolìvar P., Glaab D., Roskos H. G. (2009). J. Appl. Phys..

[cit15] Öjefors E., Lisauskas A., Glaab D., Roskos H. G., Pfeiffer U. R. (2009). J. Infrared, Millimeter, Terahertz Waves.

[cit16] Dyakonov M., Shur M. (1993). Phys. Rev. Lett..

[cit17] Lisauskas A., Boppel S., Matukas J., Palenskis V., Minkevičius L., Valušis G., Haring-Bolìvar P., Roskos H. G. (2013). Appl. Phys. Lett..

[cit18] Grzyb J., Pfeiffer U. (2015). J. Infrared, Millimeter, Terahertz Waves.

[cit19] Bauer M., Rämer A., Chevtchenko S. A., Osipov K. Y., Čibiraitė D., Pralgauskaitė S., Ikamas K., Lisauskas A., Heinrich W., Krozer V., Roskos H. G. (2019). IEEE Trans. Terahertz Sci. Technol..

[cit20] Sun J. D., Zhu Y. F., Feng W., Ding Q. F., Qin H., Sun Y. F., Zhang Z. P., Li X., Zhang J., Li X. X., Shangguan Y., Jin L. (2020). Opt. Express.

[cit21] Bauer M., Venckevičius R., Kašalynas I., Boppel S., Mundt M., Minkevičius L., Lisauskas A., Valušis G., Krozer V., Roskos H. G. (2014). Opt. Express.

[cit22] Lisauskas A. , Boppel S., Seliuta D., Minkevičius L., Kašalynas I., Valušis G., Khamaisi B., Krozer V., Socher E. and Roskos H. G., Latin America Optics and Photonics Conference, 2012, p. LM4A.1

[cit23] Ikamas K., Čibiraitė D., Lisauskas A., Bauer M., Krozer V., Roskos H. G. (2018). IEEE Electron Device Lett..

[cit24] Glaab D., Boppel S., Lisauskas A., Pfeiffer U., Öjefors E., Roskos H. G. (2010). Appl. Phys. Lett..

[cit25] Lisauskas A., Boppel S., Mundt M., Krozer V., Roskos H. G. (2013). IEEE Sens. J..

[cit26] Boppel S., Lisauskas A., Max A., Krozer V., Roskos H. G. (2012). Opt. Lett..

[cit27] Yuan H., Voss D., Lisauskas A., Mundy D., Roskos H. G. (2019). APL Photonics.

[cit28] Boppel S., Lisauskas A., Mundt M., Seliuta D., Minkevičius L., Kašalynas I., Valušis G., Mittendorf M., Winnerl S., Krozer V., Roskos H. G. (2012). IEEE Trans. Microwave Theory Tech..

[cit29] Öjefors E., Pfeiffer U., Lisauskas A., Roskos H. G. (2009). IEEE J. Solid-State Circuits.

[cit30] Hillenbrand R., Knoll B., Keilmann F. (2001). J. Microsc..

[cit31] Mastel S., Lundeberg M. B., Alonso-González P., Gao Y., Watanabe K., Taniguchi T., Hone J., Koppen F. H. L., Nikitin A. Y., Hillenbrand R. (2017). Nano Lett..

[cit32] These detectors are now becoming commercially available

[cit33] Čibiraitė-Lukenskienė D. , Lisauskas A., Ikamas K., Martín-Mateos P., de Dios Fernandez C., Acedo Gallardo P. and Krozer V., Proc. of the 23rd International Microwave and Radar Conference (MIKON), 2020, pp. 1–5

[cit34] Zhang J., Chen X., Mills S., Ciavatti T., Yao Z., Mescall R., Hu H., Semenenko V., Fei Z., Li H., Perebeinos V., Tao H., Dai Q., Du X., Liu M. (2018). ACS Photonics.

[cit35] Yao Z., Semenenko V., Zhang J., Mills S., Zhao X., Chen X., Hu H., Mescall R., Ciavatti T., March S., Bank S. R., Tao T. H., Zhang X., Perebeinos V., Dai Q., Du X., Liu M. (2019). Opt. Express.

[cit36] Babicheva V. E., Gamage S., Stockman M. I., Abate Y. (2017). Opt. Express.

[cit37] Huber A., Kazantsev D., Keilmann F., Wittborn J., Hillenbrand R. (2007). Adv. Mater..

[cit38] Tuca S.-S., Kasper M., Kienberger F., Gramse G. (2017). IEEE Trans. Nanotechnol..

[cit39] Ahmad Z., Lisauskas A., Roskos H. G., O K. K. (2019). J. Appl. Phys..

[cit40] Klimenko O. A., Knap W., Iniguez B., Coquillat D., Mityagin Y. A., Teppe F., Dyakonova N., Videlier H., But D., Lime F., Marczewski J., Kucharski K. (2012). J. Appl. Phys..

[cit41] Qin H., Li X., Sun J. D., Zhang Z. P., Sun Y. F., Yu Y., Li X. X., Luo M. C. (2017). Appl. Phys. Lett..

[cit42] Ikamas K. , Solovjovas A., Čibiraitė-Lukenskienė D., Krozer V., Lisauskas A. and Roskos H. G., Proc. of the International Conference on Infrared, Millimeter, and Terahertz Waves (IRMMW-THz), New York, NY, USA, 2020

